# Jensen’s force and the statistical mechanics of cortical asynchronous states

**DOI:** 10.1038/s41598-019-51520-2

**Published:** 2019-10-23

**Authors:** Victor Buendía, Pablo Villegas, Serena di Santo, Alessandro Vezzani, Raffaella Burioni, Miguel A. Muñoz

**Affiliations:** 1 Departamento de Electromagnetismo y Física de la Materia e Instituto Carlos I de Física Teórica y Computacional, Universidad de Granada, E-18071 Granada, Spain; 2 Dipartimento di Matematica, Fisica e Informatica, Università di Parma, via G.P. Usberti, 7/A, 43124 Parma, Italy; 3 INFN, Gruppo Collegato di Parma, via G.P. Usberti, 7/A, 43124 Parma, Italy; 4 IMEM-CNR, Parco Area delle Scienze 37/A, 43124 Parma, Italy; 5 Scuola Internazionale Superiore di Studi Avanzati, via Bonomea, 265, 34136 Trieste, Italy

**Keywords:** Biological physics, Statistical physics

## Abstract

Cortical networks are shaped by the combined action of excitatory and inhibitory interactions. Among other important functions, inhibition solves the problem of the all-or-none type of response that comes about in purely excitatory networks, allowing the network to operate in regimes of moderate or low activity, between quiescent and saturated regimes. Here, we elucidate a noise-induced effect that we call “Jensen’s force” –stemming from the combined effect of excitation/inhibition balance and network sparsity– which is responsible for generating a phase of self-sustained low activity in excitation-inhibition networks. The uncovered phase reproduces the main empirically-observed features of cortical networks in the so-called asynchronous state, characterized by low, un-correlated and highly-irregular activity. The parsimonious model analyzed here allows us to resolve a number of long-standing issues, such as proving that activity can be self-sustained even in the complete absence of external stimuli or driving. The simplicity of our approach allows for a deep understanding of asynchronous states and of the phase transitions to other standard phases it exhibits, opening the door to reconcile, asynchronous-state and critical-state hypotheses, putting them within a unified framework. We argue that Jensen’s forces are measurable experimentally and might be relevant in contexts beyond neuroscience.

## Introduction

Networks of excitatory units –in which some form of “activity” propagates between connected nodes– are successfully used as abstract representations of propagation phenomena as varied as epidemics, computer viruses, and memes in social networks^1^. Some systems of outmost biological relevance cannot be, however, modeled simply as networks of excitatory units. Nodes that inhibit (or repress) further activations are essential components of neuronal circuits in the cortex^2^, as well as of gene-regulatory, signaling, and metabolic networks^3,4^. Actually, these are an essential feature of cortical networks as synaptic excitation occurs always in concomitance with synaptic inhibition. What is the function of such a co-occurrence? or, quoting a recent review article on the subject, “why should the cortex simultaneously push on the accelerator and on the brake?”^5^.

Generally speaking, inhibition entails much richer sets of dynamical patterns including oscillations and other counterintuitive phenomena^6,7^. For example, in a nice and intriguing paper that triggered our curiosity, it was argued that inhibition induces “ceaseless” activity in excitatory/inhibitory (E/I) networks^8^. More in general, inhibition helps solving a fundamental problem in neuroscience, namely, that of the dynamic range, defined as follows. Each neuron in the cortex is connected to many others, but individual synapses are relatively weak, so that each single neuron needs to integrate inputs from many others to become active.

This leads to the existence of two alternative phases, a completely quiescent and an active/saturated one (phases are characterized by the average value of the network activity which acts as a control parameter). In other words, increasing the synaptic coupling strength leads to an explosive, all-or-none type of recruitment in populations of purely excitatory neurons when a threshold value is crossed, i.e. to a discontinuous phase transition between a quiescent and an active phase^5^. Having only two possible phases (quiescent and active/saturated) would severely constrain the set of possible network states, hindering the network capacity to produce diverse responses to differing inputs. This picture changes dramatically in the cortex, where the presence of inhibition has been empirically observed to allow for much larger dynamic ranges owing to a progressive (smoother) recruitment of neuronal populations^9,10^. This is consistent with the well-known empirical fact that neurons in the cerebral cortex remain slightly active even in the absence of external stimuli^11–13^. In such a state of low self-sustained activity neurons fire in a steady but highly-irregular fashion at a very low rate and with little correlations among them. This is the so-called *asynchronous state*, which has been argued to play an essential role for diverse computational tasks^14–17^.

It has become widely accepted that such an asynchronous state of low spontaneous activity emerges from the interplay between excitation and inhibition. Models of *balanced* E/I networks, in which excitatory and inhibitory inputs largely compensate each other, constitute –as it was first theoretically proposed^18–22^ and then experimentally confirmed^23–27^– the basis to rationalize asynchronous states. Indeed, balanced E/I networks are nowadays considered as a sort of “standard model” of cortical dynamics^28^.

In spite of solid theoretical and experimental advances, a full understanding of the phases of E/I networks remains elusive. For instance, it is still not clear if simple mathematical models can sustain highly-irregular low-activity phases even in the complete absence of external inputs from other brain regions. Indeed, many existing approaches to the asynchronous state assume that it requires of external inputs from other brain regions to be maintained^29^, while some others rely on endogenously firing neurons –i.e. firing even without inputs– for the same purpose (see e.g.^30^). Furthermore, it is not clear from modelling approaches whether asynchronous states can have very low (rather than high or moderate) levels of activity^29,31,32^.

All these problems can be summarized –from a broader Statistical Mechanics perspective– saying that it is not well-understood whether the asynchronous state constitutes an actual physical phase of self-sustained activity different from the standard quiescent and active ones. It is not clear either if novel non-standard types of phase transitions emerge at its boundaries. Such possible phase transitions might have important consequences for shedding light in to the so-called “criticality hypothesis”. This states that the cortex might operate close to the edge of a phase transition to optimize its performance. To shed light onto such a conjecture it is essential to first understand what the possible phases and phase transitions of cortical networks are.

Here, we analyze the simplest possible network model of excitatory and inhibitory nodes in an attempt to construct a parsimonious –understood as the simplest possible yet not-trivial– approach to of E/I networks^8^. We show, by employing a combination of theoretical and computational analyses, that the introduction of inhibitory interactions into purely excitatory networks leads to a self-sustained low-activity phase intermediate between conventional quiescent and active phases. Remarkably, the novel phase stems from a noise-induced mechanism that we call “Jensen’s force” (or “Jensen’s drift”) –for its relationship with Jensen’s inequality in probability theory– and that occurs owing to the combined effect of inhibition and network sparsity. The low-activity intermediate phase shares all its fundamental properties with asynchronous states and thus, as we argue, our model constitutes the simplest possible statistical-mechanics representation of asynchronous endogenous cortical activity. Moreover, continuous (critical) phase transions –separating the novel intermediate phase from the quiescent and active phases, respectively– are elucidated, with possible important consequences to shed light on the criticality hypothesis^33–35^, and to make an attempt to reconcile the asynchronous-state and criticality hypotheses, putting them together within a unified framework. Finally, we propose that the elucidated Jensen’s force might be relevant in other contexts such as e.g. gene regulatory networks.

## Models and Results

### Minimal model

**Figure 1:**
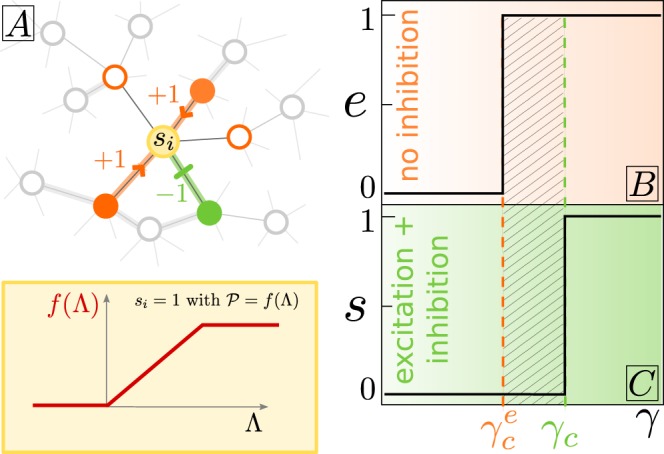
(**A**) Upper panel: Sketch of the input received by a single node, including excitatory (orange arrows) and inhibitory (green blunt arrows) interactions from active (colored) neighbors. The lower panel shows the considered transfer function for probabilistic activation of nodes as a function of the input. (**B**) Averaged level of activity in a fully-connected network consisting solely of $N(1-\alpha )$ excitatory nodes; it exhibits a discontinuous phase transition at ${\gamma }_{c}^{e}(\alpha )=1/(1-\alpha )$ separating a quiescent or Down state from an active or Up one. (**C**) As (**B**) but for a network consisting of $N(1-\alpha )$ excitatory and *Nα* inhibitory nodes. Let us remark that the shape of the phase transition depends on our choice for the transfer function. More plausible, non-linear, transfer functions lead e.g. to discontinuous transitions with a region of bistability (phase coexistence) and hysteresis; however, the main results of this work are remain unaffected (see Supplementary Information (SI) 3).

The simplest approach to capture the basic elements of E/I networks are two-state (binary) neuron models^16,18^, such as the one proposed by Larremore *et al*.^8^. The simplified version that we consider here consists of a random-regular directed network with *N* nodes and *K* links^36^. A fraction *α* of the nodes (typically $\alpha =0.2$ to mimic empirical observations^27,37^) are *inhibitory* (negative interactions) and the rest are excitatory (positive interactions). More specifically, we consider the network to be *hyper*-*regular*, meaning that not only all nodes have the same inbound and outbound connectivity $k=K/N$, but also that each of them receives exactly *αk* inhibitory inbound links and $(1-\alpha )k$ of excitatory ones (see Fig. 1 and Methods).

At any given (discrete) time *t* the state of a single node, *i*, can be either active, ${s}_{i}(t)=1$, or inactive ${s}_{i}(t)=0$. The dynamics is such that each node *i* integrates the (weighted) activity of its *k* neighbors as sketched in Fig. 1. At time $t+1$, *s*_*i*_ becomes active (resp. inactive) with probability ${\mathscr{P}}_{i}$ (resp. $1-{\mathscr{P}}_{i}$) given by

1 $$\begin{array}{l}{\mathscr{P}}_{i}\equiv f({\Lambda }_{i}=\frac{\gamma }{k}\,\sum _{j}\,{\omega }_{ij}{s}_{j}(t))=\{\begin{array}{ll}0 & {\Lambda }_{i} < 0\\ {\Lambda }_{i} & 0\le {\Lambda }_{i}\le 1\\ 1 & {\Lambda }_{i} > 1\end{array}\end{array}$$

where *f* is a transfer function of the input ${\Lambda }_{i}$, *j* runs over the set of (*k*) nodes pointing to node *i*, *ω*_*ij*_ is the weight of the connection from node *j* to node *i* (${\omega }_{ij}=\pm \,1$, for simplicity), and the control parameter *γ* is the overall coupling strength.

The model is kept purposely simple in an attempt to reveal the basic mechanisms of its collective behavior; more complex network architectures, transfer functions, and other realistic ingredients are implemented *a posteriori* to verify the robustness of the results.

### Mean-field approach: massively connected networks

We start considering the case of a fully connected network. Let *E* and *I* be the total number of excitatory and inhibitory active nodes, respectively, at a given time. These evolve stochastically according to a Master equation (as described in Methods), from which –performing a 1/*N* expansion– one readily obtains the following deterministic equations: $\dot{e}=(1-\alpha )\,\langle f(\Lambda )\rangle -e$ and $\dot{i}=\alpha \langle f(\Lambda )\rangle -i$ –where the dot stands for time derivative- for $e=E/N$ and $i=I/N$, respectively. It follows that, in the steady state, excitation and inhibition are proportional to each other: $e/(1-\alpha )=i/\alpha$, i.e. they become spontaneously balanced. Moreover, the overall activity density, $s=e+i$, obeys

2 $$\dot{s}=\langle f(\Lambda )\rangle -s,$$

while the difference $q=e-i$ is simply proportional to *s* in the stationary state: $q=(1-2\alpha )s$. In the large network-size limit ($N\to \infty$), fluctuations in the input of each node are negligible. In such a limit, all nodes receive the same input, and thus the mean-field approach, in which the mean of the transfer function values (outputs) is replaced by the transfer function of the mean input

3 $$\dot{s}=f(\langle \Lambda \rangle )-s,$$

becomes exact. Equation (3) admits two trivial fixed points corresponding to the quiescent (${s}^{\ast }=0$) and saturated (${s}^{\ast }=1$) states, respectively. The quiescent (resp. saturated) state is stable below a given value of the coupling constant, $\gamma  < {\gamma }_{c}=1/(1-2\alpha )$ (resp. $\gamma  > {\gamma }_{c}$), while right at *γ*_*c*_ all values of $0\le s\le 1$ are marginally stable. Thus, as illustrated in Fig. 1B, the system experiences a discontinuous phase transition at *γ*_*c*_ (i.e. the all-or-none phenomenon described in the Introduction). Observe also (see Fig. 1C) that, in agreement with intuition, as the fraction of inhibitory nodes in the network is increased (i.e. as *α* grows), the overall level of activity tends to decrease, and the nature of the phase transition is not altered: it remains discontinuous even in the presence of inhibitory populations.

### Beyond mean-field: Sparse networks

**Figure 2:**
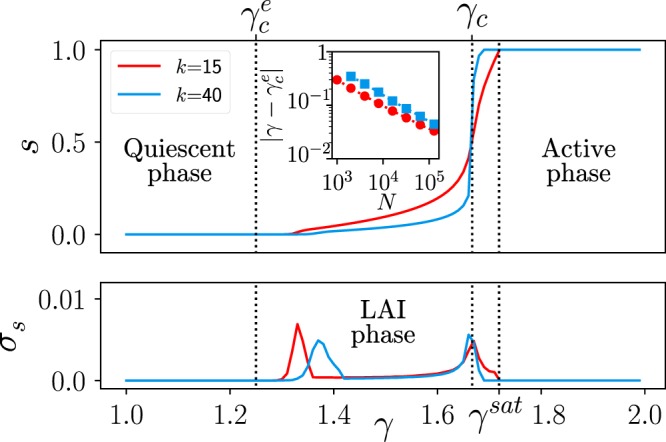
Overall steady-state averaged network activity *s* for the E/I model on a sparse hyper-regular network ($N=16,000$) in which all nodes have the same (in-)connectivity *k* (with either $k=15$ or $k=40$) and the same fraction of ($(1-\alpha )k$) excitatory and (*αk*) inhibitory inputs ($\alpha =0.2$ here). (A, Bottom) Variance across (10^3^) runs of the total network activity averaged in time windows of a given length ($T={10}^{4}$ MonteCarlo steps) as a function of the coupling strength *γ* for two different values of the connectivity *k*; each curve shows two marked peaks, indicative of two phase transitions. The leftmost one, ${\gamma }_{c}^{e}(k,N)$, shifts towards ${\gamma }_{c}^{e}$ in the large-*N* limit, obeying finite-size scaling, as illustrated by the straight line in the double-logarithmic plot of the inset. On the other hand, the second peak is a remanent of the mean-field first-order transition at ${\gamma }_{c}=1/(1-2\alpha )=1.66\ldots$ and is less sensitive to finite-connectivity effects (it is always located at the point where $s=1/2$).

Computational analyses of the model on sparse networks reveal a phenomenology much richer than the just described mean-field one. As shown in Fig. 2 the phase transition becomes progressively smoother (continuous) as the network connectivity *k* is reduced, and a novel intermediate phase where the overall average activity *s* does not saturate to either 0 or 1 emerges. Importantly, let us stress that such an intermediate phase does not appear in sparse networks of purely excitatory nodes.

To gauge the level of network-state variability, we measured the standard deviation *σ*_*s*_ of $\bar{s}$ (average of *s* finite-time windows for finite-size networks; see Fig. 2) over realizations. This quantity exhibits two marked peaks (Fig. 2) suggesting the existence of two phase transitions^38,39^. The (leftmost) peak, at ${\gamma }_{c}^{e}$, corresponds to a transition from the quiescent ($s=0$) to the *low*-*activity intermediate* (*LAI*) phase. Observe that, ${\gamma }_{c}^{e}$ exhibits severe finite-size-scaling corrections (depending also on *k*) and converges to ${\gamma }_{c}^{e}=1/(1-\alpha )$ as $N\to \infty$ (see the inset in Fig. 2). This value of *γ* coincides with the mean-field transition point for the purely excitatory subnetwork with $N(1-\alpha )$ units (i.e. without inhibition; see Fig. 1A), justifying the superindex *e* in ${\gamma }_{c}^{e}$. On the other hand, the second peak is located at ${\gamma }_{c}=1/(1-2\alpha )$, i.e. the very same location of the mean-field discontinuity for the fully-connected network and is less sensitive to finite-connectivity effects. These two transition points delimit the LAI phase. There is a third relevant value, $\gamma ={\gamma }^{sat}$ (within the active phase) at which the fully-saturated solution, $s=1$, emerges. As *k* increases, this third point becomes closer to *γ*_*c*_, making the second transition progressively sharper and converging to the mean-field result.

### Analytical results for sparse networks

To rationalize the novel intermediate-activity phase, it is essential to realize that, in the sparse-connectivity case, the input received by a given node does not necessarily take its mean-field value, but is a fluctuating variable, making it thus necessary to consider Eq. (2) rather than its mean-field counterpart Eq. (3) to correctly describe sparse networks. To make analytical progress it is necessary to determine the probability distribution of inputs, which is equivalent to computing the probability ${p}_{lj}(s)$ that a given node has exactly *l* active inhibitory neighbors and *j* active excitatory ones, for arbitrary values of *l* and *j*. Larremore *et al*. made an attempt to solve this problem working with the actual (“quenched”) network architecture, i.e. considering a specific pattern of connections between nodes. This requires analyzing the corresponding connectivity (adjacency) matrix and its leading eigenvalues^8^. Here, we propose to tackle the problem from a complementary angle. More specifically, we consider a random-neighbor (“annealed”) network version of the model, in which, at each time step, the neighbors of each node are randomly sampled from the whole network (keeping fixed the number of them as well as the fractions of excitatory and inhibitory ones). This annealed variant of the model greatly simplifies the analytical calculations, and –quite surprisingly– leads to results identical (up to numerical precision) to those for the original quenched-network problem.

For the annealed version of the model one can readily write (see SI-1):

4 $${p}_{lj}(s)=(\begin{array}{c}k\alpha \\ l\end{array})(\begin{array}{c}k(1-\alpha )\\ j\end{array}){s}^{j+l}{(1-s)}^{k-j-l},$$

which is the product of two bimodal distributions, and depends on the probability for any arbitrary node to be active, *s*. From this, it follows that

5 $$\langle f(\Lambda )\rangle ={\sum }^{}_{l,j}\,{p}_{lj}(s)f[\tilde{\gamma }(j-l)],$$

where $\tilde{\gamma }=\gamma /k$, as well as $\langle \Lambda \rangle =\gamma (1-2\alpha )s$ and ${\sigma }^{2}(\Lambda )={\gamma }^{2}s(1-s)/k$ for the mean and the variance of the input distribution, respectively. Note that all these are functions of *s* and $\tilde{\gamma }$, solely. Evaluating Eq. (5) is not straightforward owing to the non-linearity of *f*. However, analytical insight can be obtained by Taylor-expanding around either of the two trivial solutions: ${s}^{\ast }=0$ or ${s}^{\ast }=1$. Expanding around ${s}^{\ast }=0$ and keeping only leading order (linear in *s*) contributions, leads to $f(\Lambda )\simeq f(\tilde{\gamma }k(1-\alpha )s)$, which plugged into Eq. (2) implies that the solution ${s}^{\ast }=0$ loses its stability at a critical point ${\gamma }_{c}^{e}=1/(1-\alpha )$, in perfect agreement with computational observations (in the $N\to \infty$ limit). Observe that, as a consequence, the LAI phase exists for all finite connectivity values and emerges at ${\gamma }_{c}^{e}$ for all *k*, but –owing to finite-size corrections– larger and large networks are required to see it as the network connectivity is increased.

**Figure 3:**
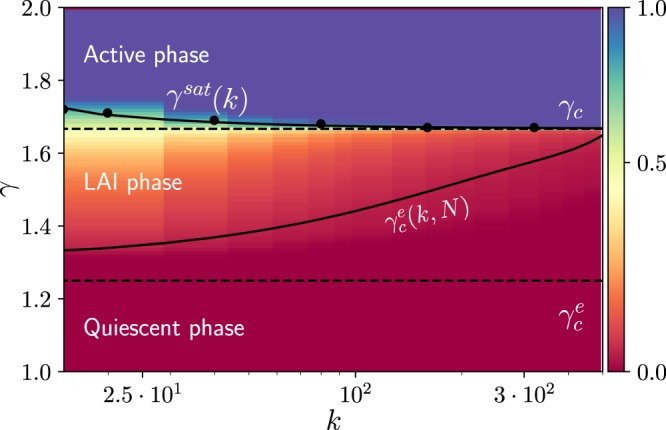
Phase diagram as a function of the coupling-strength (*γ*) and the connectivity *k* for a finite size $N=16000$ nodes. The color code indicates the level of averaged overall activity *s*; this shifts from the quiescent phase (reddish colors) to the active phase (blueish colors). Horizontal dashed lines correspond to the critical points ${\gamma }_{c}^{e}$ and ${\gamma }_{c}$ in the large-N (thermodynamic) limit. The saturation value ${\gamma }^{sat}(k)$ corresponds to Eq. (6); results from simulations are marked as black points. The curve ${\gamma }_{c}^{e}(k,N)$ represents an interpolation of the values obtained from simulations and coincides within numerical precision with the dashed line in the large-N limit.

A similar analysis around ${s}^{\ast }=1$ (see SI-2) reveals that the saturated solution is stable only above

6 $${\gamma }^{sat}=\frac{1-k(1-\alpha )}{(1-\alpha )-k(1-\alpha )(1-2\alpha )},$$

again in perfect agreement with numerical findings (see Figs 2 and 3). As numerically observed, ${\gamma }^{sat}$ converges to the mean-field prediction $1/(1-2\alpha )$ for $k\to \infty$. Thus, contrarily to mean-field expectations, there exists a whole intermediate region, ${\gamma }_{c}^{e} < \gamma  < {\gamma }^{sat}$, where activity does not vanish nor saturate for E/I networks. Such a region emerges as a consequence of input fluctuations and, hence, stems from network sparsity. Observe that for purely excitatory networks, i.e. with $\alpha =0$, ${\gamma }_{c}^{e}={\gamma }^{sat}$ and the intermediate region vanishes. The full phase diagram as a function of *γ* and *k* is depicted in Fig. 3.

### Jensen’s force

To go beyond perturbative results, note that the difference between the exact equation for the model on a sparse network, Eq. (2), and its mean-field approximation, Eq. (3), is that $\langle f(\Lambda )\rangle \ne f(\langle \Lambda \rangle )$. In other words, the non-linear function *f* and the network average are non-commuting “operators”. The reported non-trivial effects for sparse networks necessarily stem from the difference between this two different quantities:

7 $$F(\tilde{\gamma },s)\equiv \langle f(\Lambda )\rangle -f(\langle \Lambda \rangle ).$$

Observe that, as the terms in the r.h.s. depend on *s*, $F(\tilde{\gamma },s)$ can be interpreted as a state-dependent stochastic “force”^40^. As shown above the distribution of inputs to any given node is centered at $\langle \Lambda \rangle$ and has a standard deviation that scales as $1/\sqrt{k}$ (in agreement with the central limit theorem). If *f* was a linear function, then $\langle f(\Lambda )\rangle =f(\langle \Lambda \rangle )$, but as it is a convex function near the origin, then the Jensen’s inequality of probability theory (this expresses the fact that if *x* is a random variable and $g(x)$ is a convex function, then $\langle g(x)\rangle \ge g(\langle x\rangle )$) implies that $\langle f(\Lambda )\rangle  > f(\langle \Lambda \rangle )$, i.e. *F* is positive if $\langle \Lambda \rangle$ is near 0, i.e. if the level of activity *s* is relatively small.

**Figure 4:**
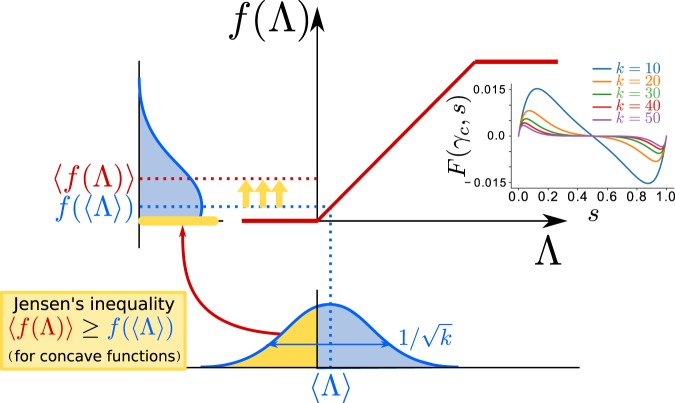
Sketch illustrating the origin of the noise-induced Jensen’s force. Each node in a sparse network receives an input $\Lambda$ which is a random variable extracted from some bell-shaped probability distribution function $P(\Lambda )$ (sketched below the x-axis) with averaged value $\langle \Lambda \rangle =\gamma (1-2\alpha )s$ and standard deviation ${\sigma }_{s}=(\gamma \sqrt{s(1-s)})/\sqrt{k}$ (see SI-1). The possible outputs $f(\Lambda )$ are also distributed according to some probability (sketched to the left of the y-axis). Given that around $\Lambda \approx 0$ the function $f(\Lambda )$ is locally convex then, as a consequence of Jensen’s inequality for convex functions, $\langle f(\Lambda )\rangle \ge f(\langle \Lambda \rangle )$ (i.e. the dotted red line is above the blue one). Indeed, while for positive inputs, the transformation is linear, negative ones are mapped into 0 thus creating a net positive Jensen’s force for small values of $\Lambda$ (or *s*). The inset shows the Jensen’s force $F(\tilde{\gamma },s)\equiv \langle f(\Lambda )\rangle  > -\,f(\langle \Lambda \rangle )$ computed right at the critical point *γ*_*c*_ for different connectivity values, as a function of *s*. Note, the negative values for large values of *s* which stem from the concavity of the function *f*(*x*) around *x* = 1. Note that *F* decreases as *k* grows and vanishes in the mean-field limit.

Thus, we propose the term “*Jensen’s force*” to refer to $F(\tilde{\gamma },s)$ (see Fig. 4). This positive force is responsible for the destabilization of the quiescent state and the emergence of the LAI phase. Observe that if, on the other hand, $\langle \Lambda \rangle$ happens to be close to 1, the function *f* is locally concave and, using a reverse argument, $\langle f(\Lambda )\rangle  < f(\langle \Lambda \rangle )$, i.e. there is a negative Jensen’s force *F* in the regime of very large activities (justifying the reduction of the saturated regime with respect to the mean-field case). Finally, if parameters are such that the system lies in the quiescent ($s=0$) or in the saturated ($s=1$) phase then there are no input fluctuations –i.e. the input distribution is delta function– and the Jensen’s force vanishes.

The Jensen’s force, $F(\tilde{\gamma },s)$, can be analytically calculated for some particular transfer functions *f* (see SI-3) but, in general, it can only be determined numerically. For the sake of illustration, results for the function *f* considered in Eq. (1) for the particular case $\gamma ={\gamma }_{c}$ are shown in the inset of Fig. 4. Observe that $F(\tilde{\gamma },s)$ is positive for $s < 1/2$, negative for $s > 1/2$ and vanishes at $s=1/2$ explaining why the steady state is precisely $s=1/2$ for $\gamma ={\gamma }_{c}$. Similar arguments work for other values of *γ*. Let us emphasize that the magnitude of the force decreases as *k* grows (Fig. 4, inset) vanishing in the limit in which networks are no longer sparse.

Summing up, the sparsity-induced Jensen’s force *F* is responsible for the emergence of a LAI phase in E/I networks below the mean-field critical point, *γ*_*c*_ as well as for a reduction in the overall level of activity with respect to the mean-field limit in a region above *γ*_*c*_.

Let us emphasize that the annealed-network approximation fits perfectly well all computational results obtained for quenched networks, with fixed neighbors and intrinsic structural disorder (we have verified that, indeed, the quenched and the annealed versions of the model give numerically indistinguishable results; see SI-3). The reason for this agreement, lies in the absence of node-to-node correlations within the LAI phase in the large-network limit (see below), which suggests that the annealed approximation is exact in such a limit.

Importantly, we have computationally verified that the LAI phase is quite robust as it also emerges for other non-linear transfer functions, more random (non hyper-regular) networks as well as for heterogeneous weight distributions (see SI-3).

### Phase transitions from and to the LAI phase

Figure 2 reveals the existence of two phase transitions, one at each side of the LAI phase. Around the left-most one, at ${\gamma }_{c}^{e}$, we performed standard computational analyses of avalanches, by introducing a single seed of activity (one active excitatory node) in an otherwise quiescent state, and analyzed the statistics of the cascades of activations it triggers.

**Figure 5:**
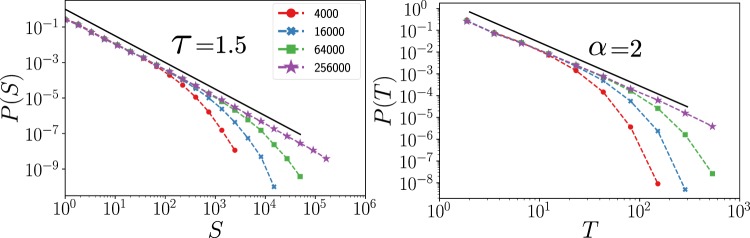
Distribution of avalanche sizes (left) and durations (right) at the (leftmost) critical point ${\gamma }_{c}^{e}$ for different system sizes (see legend) in a hyper-regular network with $k=15$. Black dotted lines are guides to the eye showing the theoretical values for an unbiased branching process.

We observed computationally that at the quiescent-active critical point ${\gamma }_{c}^{e}$ the system displays avalanches –whose sizes and durations are distributed as power-laws as $P(S)\sim {S}^{-\tau }$ and $P(T)\sim {T}^{-\alpha }$, respectively (see Fig. 5)– thus compatible with those of the unbiased branching process^41,42^. This result is not surprising given the un-structured (mean-field like) nature of the network. Further analyses need to be done in lower dimensional systems to see if this transition from a quiescent to a noise-induced active phase shares the critical features of standard quiescent-to-active phase transitions (known to be in the so-called directed percolation universality class^39,43^) or if novel behavior emerges owing to noise-induced effects. In any case, our simple model does not exhibit correlations between succesive avalanches, which are often observed in experimental settings^44^. On the other hand, the second phase transition, at ${\gamma }_{c}=1/(1-2\alpha )$ is a remanent of the original (discontinuous) mean-field one, and signals a (continuous) transition between states of low activity to high activity ones. This phase transition –which is driven by the Jensen force that changes from positive to negative, i.e. it vanishes, at the transition point– also needs further scrutiny to be fully elucidated. A detailed analysis of these phase transitions, as well as of their possible relevance in connection with the hypothesis that the cerebral cortex might operate at the edge of a critical point^34,35,41,45,46^ is left as an open challenge for future work (see Discussion).

### Asynchronous-state features

The cortical *asynchronous* state is characterized by a number of key features (see also Methods) including: (**i**) Large variability: the coefficient of variation, *CV*, defined as the ratio of the standard deviation to the mean of the interspike intervals (i.e. periods of un-interrupted silence for a given neuron/node) is relatively large, i.e. *CV* ≥ 1^11^. (**ii**) The network-averaged pairwise Pearson’s correlation coefficient *PC* is very low; actually it decays to 0 with network size reflecting a lack of synchronization or coherent behavior^22,29,47^. (**iii**) There is a (short) time lag between excitation and inhibition (E-I lag) meaning that an excess in excitation is rapidly compensated by an increase in inhibitory activity, so that inhibition actively de-correlates neural populations and the network state remains stable, as theoretically predicted^22,48–50^ and experimentally confirmed^14,51^.

**Figure 6:**
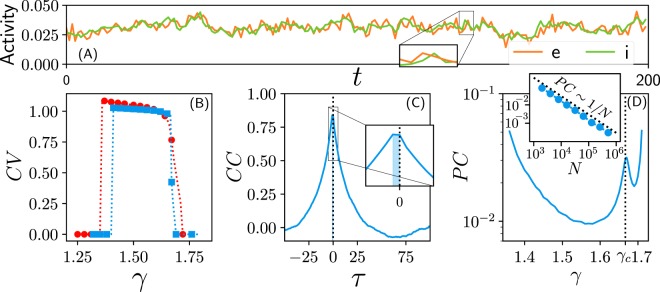
(**A**) Time series of the excitatory (e; orange line) and inhibitory (i; green line) network activity in the LAI phase (network $N=16000$). The zoom illustrates the small (one-time step) E-I lag present in this phase. (**B**) Coefficient of variation (CV) vs. coupling-strength *γ*; $CV\ge 1$ within the LAI phase, while it vanishes in the quiescent and active phases (the color code, as in Fig. 2, stands for connectivity values). (**C**) Time-lagged cross-correlation (CC) between the excitation and inhibition timeseries in the LAI phase. The maximum (black dashed line) reflects the existence of a one-step E-I lag. (**D**) Pairwise Pearson’s correlation (PC) between nodes in the LAI phase as function of *γ*; it takes small values, but exhibits a marked peak at the critical point *γ*_*c*_ (dotted line). The inset shows that the PCs scale with system size as 1/*N* thus vanishing in the large-network limit (data for $\gamma =1.55$, but results valid all across the LAI phase). In all cases, we considered enough simulation runs so that errorbars are smaller than the employed symbols. Cross-Correlation and E-I lag have been obtained from a raster of $N=16000$ neurons for $t={10}^{4}$. The pairwise correlation is computed taking 500 random pairs, averaging over 1000 different networks.

As shown in Fig. 6 the LAI phase –but not the quiescent nor the active ones– displays all these key features of cortical asynchronous states (see figure caption for details). In particular, the coefficient of variation CV is larger than unity all across the LAI phase, while it vanishes in the quiescent and saturated phases. The value of CV could be made even larger –so that larger levels of variability would be obtained, mimicking more closely experimental results (see e.g.^44^) by introducing more complex network topologies and/or more complex gain stochastic gain functions^52,53^. Also, there is a one-step time-lag correlation between excitation and inhibition, revealing that the population of inhibitory neurons lags behind following and controlling the level of excitatory activity. Finally, the correlation between any pair of neurons (regardless of whether each of them is excitatory or inhibitory) is very small all across the LAI phase and tends to vanish in the large network-size limit, implying that pairs of neurons become decorrelated.

**Figure 7:**
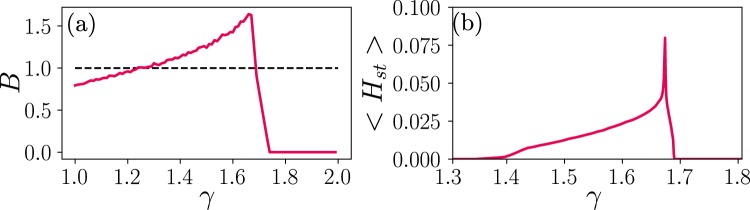
(**a**) Branching function *B* in damage spreading experiments (averaged over 10^4^ runs). Black dotted lines represent marginal propagation of activity, i.e. critical dynamics. All across the LAI phase, the dynamics propagates in a chaotic way, *B* > 1, while in the quiescent and active phases, the Hamming distance is smaller than 1. (**b**) Average over runs for the time-averaged Hamming distance in the steady state $\langle {H}_{st}\rangle$, over $T={10}^{4}$ MonteCarlo steps; two initial replicas are different in a small number (10) of nodes. In this case, all across the LAI phase the difference between the two replicas $\langle {H}_{st}\rangle$ is very close to the steady state density, indicating that activity becomes uncorrelated between them (node states coincide only by chance). Simulations run for hyper-regular networks with $N=16,000$, $k=40$ and $\alpha =0.2$.

Moreover, in agreement with the original claim for asynchronous states^18,19^, we verified that all along the LAI phase (and only in the LAI phase) the dynamics is chaotic (or quasi-chaotic) in the sense of damage spreading dynamics^54^. Indeed, as shown in Fig. 7a, all across the LAI phase we observe a value of the branching parameter $B > 1$ and, as a consequence, chaotic behavior, as previously suggested for asynchronous states^18^ (see Methods). Moreover, by computing the difference between states in *M* and *M*′ that differ in a few sites, and computing the averaged Hamming distance for sufficiently large times (*H*_*st*_), one observes that such a distance takes values of the order of the network activity within the LAI phase (see Fig. 7b), revealing that active sites become rapidly uncorrelated in both replicas and reflecting again the chaotic nature of the LAI phase.

Thus, in synthesis, all the chief features of cortical asynchronous states are also distinctive and exclusive characteristics of the LAI phase.

### Tightly-balanced networks

We now scrutinize how the region in parameter space in which the LAI phase emerges can be maximized, thus limiting the need for parameter fine tuning to exploit the possible functional advantages of such a regime. This is achieved by considering *tightly*-*balanced* networks (also called detailed-balanced networks)^17,55^ in which excitatory and inhibitory inputs are tuned to compensate mutually, so that the average input of individual nodes is kept close to 0. To do so, it suffices to introduce in the model definition, Eq. (1), two different strengths for excitatory and inhibitory synapses, ${\omega }^{e}$ and ${\omega }^{i}$, respectively. In this way, the (leftmost) transition point is easily seen to shift to, ${\gamma }_{c}^{e}=1/({\omega }^{e}(1-\alpha ))$, while *γ*_*c*_ changes to

8 $${\gamma }_{c}=\frac{1}{({\omega }^{e}(1-\alpha )-{\omega }^{i}\alpha )}$$

which diverges to infinity if ${\omega }^{e}/{\omega }^{i}=\alpha /(1-\alpha )$. This implies that the largest possible LAI phase is obtained when this latest condition is met (observe that in such a limit the level of activity varies very slowly converging to $s=1/2$ as $\gamma \to \infty$). Given that as shown above $e/(1-\alpha )=i/\alpha$, the above condition corresponds precisely to the tightly-balanced networks for which the averaged input of each single node, $\langle \Lambda \rangle =\tilde{\gamma }({\omega }^{e}e-{\omega }^{i}i)$, vanishes. Thus, tightly-balanced networks have the largest possible LAI phase and the largest possible dynamic range.

### Experimental measurements of the LAI phase and the Jensen’s force

Is it possible to measure the Jensen’s force experimentally? We believe it is, but explicitly designed setups would be required. First of all, let us recall that asynchronous states (i.e. LAI phases) have been detected experimentally both *in vivo* and *in vitro*^24,25,27^. Importantly, with today’s technology, the spiking activity of more than 1000 neurons can be measured simultaneously^56^, so that much better statistics can be collected. In principle, one should be able to compute the Jensen’s force in this type of experiments. In SI-4 we propose a tentative experimental protocol to do so. However, we leave this programme for future research as well as an open challenge for experimentalists.

## Conclusions and Discussion

It has been long observed that neurons in the brain cortex remain active even in the absence of stimuli^11–13^. Depending mostly on cortical region and functional state, diverse levels of synchronization across the asynchronous-synchronous spectrum are observed^20,30^. While the role of synchronization in neuronal networks has been long studied^57^, the role of the asynchronous state remained more elusive^22^. Presently, it has become widely accepted that the asynchronous state emerges from the interplay between excitation and inhibition, and that it is essential for network stability and to allow for high computational capabilities^14–16^.

Our main goal here was to investigate the origin of low-activity regimes in excitation/inhibition networks, determining in particular the nature of their (thermodynamic) phases. For this, we employed a statistical-mechanics approach and searched for a model as parsimonious as possible, i.e. a sort of Ising model of E/I networks. In particular, we analyzed a model which further simplifies the one proposed by Larremore *et al*.^8^ in a few different ways. For example, we removed network heterogeneity both in its architecture and in the allowed synaptic weights to allow for mathematical tractability.

Our main result is that E/I networks exhibit a non-trivial LAI phase in between standard quiescent and active phases, in which activity reverberates indefinitely without the need of external driving, nor of intrinsically firing neurons (in contradictions to previous beliefs). Two key ingredients are necessary for the LAI phase to emerge: a spontaneously generated dynamical balance between excitation and inhibition and network sparsity. The resulting phase has all the statistical properties usually ascribed to asynchronous states.

The LAI phase stems from network sparsity, i.e. from fluctuations in input values and appear even in extremely homogeneous, hyper-regular, networks. This observation disproved an initial conjecture of us suggesting that intermediate levels of activity could be related to so-called *Griffiths phases*. Such phases have remarkable features^58^ and have been claimed to be relevant for cortical dynamics^59^; they also emerge in between quiescent and active phases, but only in systems characterized by structural heterogeneity and, thus, are unrelated to the novel LAI phase uncovered here. Nevertheless, an important research line left for future work, is to analyze how the properties of the LAI phase are altered in more structured and realistic networks including e.g. broad degree distributions, clustered structure and modular-hierarchical organization, which might lead to novel phenomena^59–62^.

An issue worth discussing is the dependence of the presented phenomena on network connectivity and the connection of our work with the standard view of balanced networks as originally proposed in the seminal work of van Vreeswijk and Sompolinski^18^. As we showed, the LAI phase emerges out of input fluctuations and –as the input standard deviation scales with $\gamma /\sqrt{k}$– it relies crucially on the finiteness of *k*, i.e. on network sparsity. However, it is important to underline that, as we showed, the LAI phase survives even for arbitrarily large values of *k*, but larger and larger network sizes *N* are required for it to be evident. Interestingly, it is also possible to adopt the original scaling proposed in^18^, where it was argued that if the strength of individual synapses is of order $1/\sqrt{k}$ (rather than constant as here), it compensates fluctuations in the number of actual inputs (order $\sqrt{k}$), leading a total input fluctuations of the same order of the neuron firing threshold (order unity), and thus to fluctuation-controlled activations. This type of scaling can be easily accommodated within our approach just by replacing *γ*/*k* in Eq. 1 by $\gamma ^{\prime} /\sqrt{k}$; with this scaling, the critical points in terms of the new coupling constant *γ*′ are shifted as *k* grows, and the noise-induced phase persists even in the limit of dense networks. Note also that, as illustrated here, having a sharp threshold is not a necessary ingredient for the phenomenon to occur: the LAI phase also emerges when considering, e.g. a transfer function such as the hyperbolic-tangent without a hard discontinuity. In other words: the Jensen’s force is more general than a hard threshold, noise-filtering, mechanism.

In order to verify whether more realistic neuronal networks models exhibit also an intermediate phase, in between quiescent and standard active ones, we first scrutinized the recent literature. We found that there are (at least) two recent computational analyses of E/I networks of integrate-and-fire neurons with (current-based or conductance-based) synapses confirming the emergence of a similar self-sustained intermediate regime with high variability^31,32^. This confirms that the very general mechanism put forward here also applies to more detailed/complicated neuron models. Observe, however, that these studies use integrate-and-fire neurons with an activation threshold, meaning that low inputs are suppressed. As a consequence, they require the use of strong synapses to trigger the Jensen’s force and induce a stable LAI. Let us, thus, emphasize that the concept of Jensen’s force sheds new light on the computational findings of these recent works.

We have proposed a tentative protocol to challenge experimentalist to empirically measure Jensen’s forces in actual neuronal networks, either *in vivo* or *in vitro*. Even if technical difficulties are likely to emerge, we strongly believe that Jensen’s forces are susceptible to be observed and quantified in the lab. This research programme, if completed, would strongly contribute to shedding light on the noisy dynamics of cortical networks, as well as on the way it helps processing information.

Let us also comment on the relationship between the so called “criticality hypothesis” –i.e. the idea that the cortex, as well as some other biological systems, might extract important functional advantages from operating near the critical point of a continuous phase transition^33–35,41,45,63^– and the findings in this work. Let us emphasize that asynchronous states and critical states have almost opposite features: the first is characterized by active de-correlation of nodes and the second exhibits strong system-spanning correlations. Thus, clarifying the interplay between these two antagonistic interpretations/phenomena –and analyzing them together within a unified framework– is a challenging goal^64,65^. We believe that our simple model (probably improved with further important ingredients such as some for of synaptic plasticity (as e.g. in^35^)) is a good candidate to constitute a unified framework to put together asynchronous and synchronous states and the critical phase transition in between, and to analyze these fundamental questions. Observe in particular that the LAI phase is separated from the quiescent and active phases, respectively, by continuous phase transitions –including critical points– whose specific details still need to be further elucidated. As a matter of fact, having a good understanding of the main phase transitions of E/I networks is a fundamental preliminary step to make solid progress to contribute to the criticality hypothesis. It is also worth mentioning that some recent papers^66^ claim that critical dynamics occurs at the network level in concomitance with the strongest detailed E/I balance at the neuronal level; indeed, as our work shows, at the limiting points of the LAI phase there is E/I balance and criticality.

Finally, let us mention that we are presently exploring the possibility of observing similar LAI phases in other biological networks such as gene regulatory ones, where gene repression plays a role equivalent to synaptic inhibition in neural networks where opposite conflicting influences may mutually compensate to each other, leading to noise-induced phenomena. We hope that the novel stochastic force and phase elucidated here foster new research along and this and similar lines.

## Methods

Some of the most relevant methods have been sketched in the main text. Here we detail some important methodological aspects. Further details are provided in the Supplementary Information (SI).

### Hyper-regular networks

**Figure 8:**
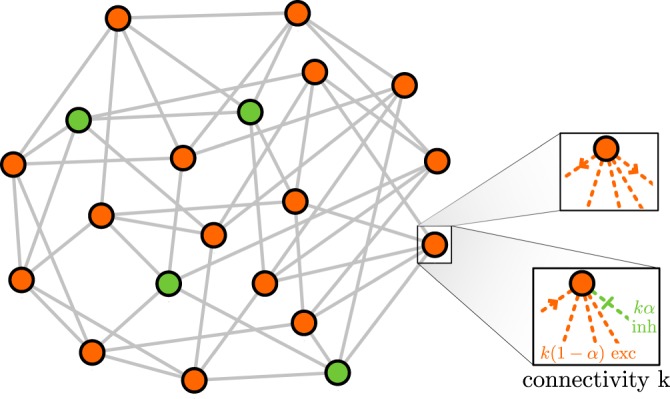
Sketch of a hyper-regular network with $N=20$ nodes and connectivity $k=5$. Orange nodes stand for excitation and green nodes for inhibition. For the zoomed node, the difference between out-activity and in-activity is also shown (i.e. each node has $k=5$ excitatory (or inhibitory) outbound links as well as $k(1-\alpha )$ excitatory and *kα* inhibitory inbound links). In particular, in this example, each node has 5 inbound inputs of which 4 are excitatory and 1 inhibitory, as well as 5 outbound links: all of them positive for excitatory units and negative for inhibitory ones.

For the sake of mathematical tractability, we consider hyper-regular networks in which each node has exactly ${k}_{exc}=k(1-\alpha )$ excitatory neighbors and ${k}_{inh}=k\alpha$ inhibitory ones pointing to it (where *α* is the fraction of inhibitory nodes); a sketch is shown in Fig. 8. For this, we follow these steps: (i) two random regular networks, one of excitatory nodes with connectivity *k*_*exc*_ and one of inhibitory units with connectivity *k*_*inh*_ are generated; (ii) ${k}^{e}=k(1-\alpha )$ links (avoiding node repetitions) are randomly chosen to point to each inhibitory node. This process got sometimes stuck due to a topological conflict, so we re-started the process after 10^6^ unsuccessful attempts to include new links. Each link of the so constructed networks is taken with positive weight for interactions from a excitatory nodes *j* to a inhibitory neuron *i* (${\omega }_{ij} > 0$) and negative for the opposite interaction (${\omega }_{ji} < 0$). On the other hand, all weights with the excitatory (resp. inhibitory) subnetwork are positive (resp. negative).

### Mean field approach

The excitatory and inhibitory populations (E,I) evolve stochastically according to a Master equation^40^ described by the following transition rates for large networks:

9 $$\begin{array}{rcl}\Omega \,(E,I\to E+1,I) & = & [N(1-\alpha )-E]\,\langle f({\Lambda })\rangle \\ \Omega \,(E,I\to E-1,I) & = & E\,[1-\langle f({\Lambda })\rangle ]\\ \Omega \,(E,I\to E,I+1) & = & [N\alpha -I]\,\langle f({\Lambda })\rangle \\ \Omega \,(E,I\to E,I-1) & = & I\,[1-\langle f({\Lambda })\rangle ]\end{array}$$

where the timescale has been set to unity, $\langle f(\Lambda )\rangle$ is the average probability for any given node to become active (〈·〉 stands for network average), and factors such as $N(1-\alpha )-E$ (resp. $(N\alpha -I)$) describe the number of inactive excitatory (resp. inhibitory) nodes. Performing a 1/*N* expansion of the corresponding Master equation^40^ and keeping terms up to leading-order, one readily obtains the following deterministic equations:

10 $$\begin{array}{rcl}\dot{e} & = & (1-\alpha )\,\langle f(\Lambda )\rangle -e\\ \dot{i} & = & \alpha \,\langle f(\Lambda )\rangle -i\end{array}$$

where the dot stands for time derivative for $e=E/N$ and $i=I/N$, respectively. In particular, considering a fully-connected system in the large size limit (i.e. $N\to \infty$), fluctuations in the input of each node are negligible. Thus, all nodes receive the same input, and the mean of the transfer function values is replaced by the transfer function of the mean input, i.e. the mean-field approach implies

11 $$\langle f(\Lambda )\rangle =f(\langle \Lambda \rangle )$$

The detailed procedure to compute these averages is presented in SI-1 and SI-2.

### Asynchronous-state features

#### Coefficient of variation (CV)

This is defined as the quotient of the standard deviation *σ*_*ISI*_ to the mean *μ*_*ISI*_ of the inter-spike interval (*ISI*) on individual units:

12 $$CV=\frac{{\sigma }_{ISI}}{{\mu }_{ISI}}.$$

#### Excitatory/inhibitory cross-correlation

Given two time series *x*(*t*) and *y*(*t*), the Pearson correlation coefficient of *x*(*t*) and $y(t+\tau )$

13 $$CC(\tau )=\frac{1}{{\sigma }_{x}{\sigma }_{y}}\,\mathop{\sum }\limits_{t=-\infty }^{+\infty }\,\overline{x(t)y(t+\tau )}.$$

where *σ*_*x*_ and *σ*_*y*_ are the standard deviations of the time series *x*(*t*) and *y*(*t*), respectively and $\tau$ is a time delay. Since we are interested in the E/I lag, we subtract the mean from the time series, i.e. we take $x(t)=e(t)-{\mu }_{e}$ and $y(t)=i(t)-{\mu }_{i}$. This procedure ensure us a correct normalization, so $CC(\tau )\in [\,-\,1,1]$. In this way, if $CC(\tau )$ has a peak for $\tau  < 0$, we conclude that the activity of the inhibitory population resembles the activity of the excitatory one, but it is shifted to the left: excitatory population spikes first and it is followed by the inhibitory one.

#### Pairwise correlation

The Pearson’s correlation coefficient between a randomly selected pair of network nodes, *x*_*i*_ and *x*_*j*_, is defined as

14 $$P{C}_{{x}_{i},{x}_{j}}=\frac{\langle {x}_{i}{x}_{j}\rangle -\langle {x}_{i}\rangle \,\langle {x}_{j}\rangle }{\sqrt{\langle {x}_{i}^{2}\rangle -{\langle {x}_{i}\rangle }^{2}}\sqrt{\langle {x}_{j}^{2}\rangle -{\langle {x}_{j}\rangle }^{2}}}$$

where 〈·〉 represents a temporal average. The total Pearson’s correlation coefficient (*PC*) is computed by averaging $P{C}_{{x}_{i},{x}_{j}}$ over 500 pairs of nodes for different realizations.

#### Chaotic behavior

In order to scrutinize the possible chaotic nature of the LAI phase^18^, we employ the standard method consisting in analyzing the dynamics of damage spreading^54^. It involves the next steps: (1) take a specific state of a network, *M*, and a construct an identical replica of it, *M*′, in which the state of only a randomly-chosen node is changed; (2) the Hamming distance, *H* –defined as the difference of states between *M* and *M*′– is computed after one time step (i.e. an update of all the nodes of the two networks) and finally, (3) *H* is averaged over many realizations (i.e. over different locations of the initial damage and stochastic trajectories) obtaining the *branching parameter*, *B*. If $B < 1$ perturbations tend to shrink and the network is in a ordered phase, while if $B > 1$ perturbations growth on average and the network exhibit chaotic-like behavior. For marginal propagation of perturbations, $B=1$, the network is critical.

## Supplementary information

Supplementary information: Jensen’s force and the statistical mechanics of cortical asynchronous states
