# Supplementary material for: Jensen’s force and the statistical mechanics of cortical asynchronous states
Source: Sci Rep. 2019 Oct 23;9:15183. doi: 10.1038/s41598-019-51520-2 (PMC6811577; doi:10.1038/s41598-019-51520-2)
Supplement: Supplementary file 1 — Supplementary information: Jensen’s force and the statistical mechanics of cortical asynchronous states [file 41598_2019_51520_MOESM1_ESM.pdf]

Supplementary information: Jensen's force and the statistical mechanics of cortical asynchronous states

Victor Buendía[1,2,3], Pablo Villegas[1], Serena di Santo[5], Alessandro Vezzani[2,4], Raffaella Burioni[2,3], and Miguel A. Muñoz[1,2,*]

[1]Departamento de Electromagnetismo y Física de la Materia e Instituto Carlos I de Física Teórica y Computacional. Universidad de Granada, E-18071 Granada, Spain
[2]Dipartimento di Matematica, Fisica e Informatica, Università di Parma, via G.P. Usberti, 7/A - 43124, Parma, Italy
[3]INFN, Gruppo Collegato di Parma, via G.P. Usberti, 7/A - 43124, Parma, Italy
[4]IMEM-CNR, Parco Area delle Scienze 37/A - 43124 Parma, Italy
[5]Scuola Internazionale Superiore di Studi Avanzati, via Bonomea, 265 - 34136 Trieste, Italy.

Contents

1 Distribution of the input of an individual neuron for annealed networks 2
2 Derivation of the critical and saturation points 2
3 Robustness of the results 3
4 Experimental measurement of Jensen's force 9
References 9

1 Distribution of the input of an individual neuron for annealed networks

Here we discuss in detail the derivation of the distribution of inputs received by each individual node within the annealed version of the model. If node has j excitatory active neighbors and l inhibitory active ones, its input is given by $\Lambda_{jl} = \tilde{\gamma}(j-l)$, with $\tilde{\gamma} = \gamma/k$. At each timestep any given node chooses randomly k connecting nodes: n inhibitory and $k-n$ excitatory ones. Thus, the probability to have an input of value Λ_{jl} is controlled by the probability distribution of having j excitatory and l inhibitory active neighbors $p(\Lambda_{jl}|n) = p(j|k-n)p(l|n)$. If the average activity of the network is s, a node picked randomly will be active with probability s, regardless of whether it is excitatory or inhibitory. As a consequence, the probability of finding l active inhibitory neighbors out of the total n inhibitory connections is given by the binomial distribution:

$$p(l|n) = \binom{n}{l} s^l (1-s)^{n-l}. \tag{1}$$

Similarly, the probability $p(j|n)$ to have a j excitatory active nodes out of $k-n$ possible ones is another binomial distribution. In the case of the hyper-regular model, in which we the number of inhibitory neighbors can be written as $n = k\alpha$ one readily obtains

$$\begin{aligned} p(\Lambda_{jl}) &= \sum_n \delta_{n,k\alpha} p(\Lambda_{jl}|n) = \\ &= \binom{k\alpha}{l} \binom{k(1-\alpha)}{j} s^{j+l} (1-s)^{k-j-l}, \end{aligned} \tag{2}$$

(while for regular but not hyper-regular networks this expresion becomes more involved). Note that the above probability depends exclusively on the average activity of the network, s, and the connectivity k, so $p(\Lambda_{jl}) \equiv p_{lj}(s)$, as defined in the main text. From this, it is possible to evaluate the averages of any function of the input. In particular, the first and second moments of the input can be easily computed

$$\langle \Lambda \rangle = \sum_{j=0}^{k-n} \sum_{l=0}^{n} p_{lj}(s) \Lambda_{jl} = \tilde{\gamma} ks(1-2\alpha) = \gamma s(1-2\alpha)$$

$$\langle \Lambda^2 \rangle = \tilde{\gamma}^2 \left[ks^2 \left((1-2\alpha)^2 k - 1 \right) + ks \right],$$

and, similarly, for the variance

$$\sigma^2(\Lambda) = \tilde{\gamma}^2 ks(1-s) = \gamma^2 s(1-s)/k \tag{3}$$

This implies that fluctuations of the input –as measured by the standard deviation σ_s– are proportional to $1/\sqrt{k}$, as expected from the central limit theorem[1,2]. Also, it follows that the states ($s=0$ and $s=1$) exhibit no fluctuations, and that the maximum level of fluctuations occurs at $s = 1/2$, which coincides with the value of the activity at the critical point γ_c.

Moreover, we assumed that each node has k inbound connections and a fixed number n of inhibitory neighbors, but, as a matter of fact, it is also possible to consider the non-regular network case by letting k to be a random variable itself, distributed according to some arbitrary probability distribution $g(k)$. This generalization changes Eq.(2) to

$$\begin{aligned} p(\Lambda_{jl}) = \sum_{k=1}^{+\infty} \sum_{n=0}^{k} \binom{n}{l} \binom{k-n}{j} g(k) \cdot \\ \cdot h(n|k) s^{j+l} (1-s)^{k-j-l} \end{aligned} \tag{4}$$

where $h(n|k)$ is the probability of having n inhibitory neighbors, given a connectivity k. Although the sum cannot be worked out analytically, it is still possible to work it out numerically. In particular, letting $g(k)$ to be a Poisson distribution and $h(n|k)$ a binomial distribution with probability α, it is possible to obtain results for Erdős-Rényi networks.

2 Derivation of the critical and saturation points

Considering the distribution of possible inputs for each node in the annealed approximation, it is straightforward to derive the critical and saturation points of the system. The equation for the activity reads

$$\dot{s} = \langle f(\Lambda) \rangle - s. \tag{5}$$

Given Eq.(2) it is possible to compute exactly the average value $\langle f(\Lambda) \rangle$:

$$\langle f(\Lambda) \rangle = \sum_{j=0}^{k-n} \sum_{l=0}^{n} f(\tilde{\gamma}(j-l)) p_{lj}(s), \tag{6}$$

where n is the number of inhibitory neighbors of a node, and j and l represent the number of active excitatory and inhibitory neighbors, respectively. Although for the hyper-regular case $n = k\alpha$, we consider here a generic n value and replace it by its value at the end of the calculation. Due to the non-linearity of the function f, it is not possible to fully solve the problem analytically. However, it is still possible to derive relevant information from equation (6). First of all, near the critical point γ_c^e, one expects to have a very low level of activity, which suggests to expand Eq.(6) up to first order in s. The probability $p_{lj}(s)$ contains a factor $s^{j+l}(1-s)^{k-j-l}$, so that, if k is large enough (and taking into account that for $\alpha = 0.2$ we should assume $k > 5$ here), all terms with $j + l \geq 2$ contribute to second order in s. Thus, the (j,l) pairs that contribute to first order are just $(0,0)$, $(0,1)$ and $(1,0)$. Of these terms, note that $f(\Lambda_{00}) = f(\Lambda_{01}) = 0$, so, the only contributing one is $(1,0)$. Taking this value in Eq.(6) and performing the Taylor expansion, one readily obtains

$$\langle f(\Lambda)\rangle = f(\tilde{\gamma})\binom{n}{0}\binom{k-n}{1}s\,(1-s)^{k-1} \simeq$$
$$\simeq f(\tilde{\gamma})k(1-\alpha)s. \tag{7}$$

Considering also that $f(\tilde{\gamma}) = \tilde{\gamma}$, we obtain

$$\dot{s} = \gamma(1-\alpha)s - s, \tag{8}$$

that exhibits a bifurcation at $\gamma_c^e = 1/(1-\alpha)$ separating the quiescent phase ($s = 0$) from the LAI phase ($s \neq 0$).

In order to obtain information about the saturation of the activity one can use the very same procedure to expand around $s = 1$. Observe that now the three pairs that contribute to first order in $(1-s)$ are $(k-n,n)$, $(k-n-1,n)$ and $(k-n,n-1)$, and $f(\Lambda_{jl})$ does not vanish for any of these terms. Introduce such values in Eq.(6), and after Taylor expanding, one finds:

$$\begin{aligned}\langle f(\Lambda)\rangle = {} & f\left[\tilde{\gamma}(k-2n)\right]\left[1+k(1-s)\right]+\\ & +(1-s)(k-n)f\left[\tilde{\gamma}(k-2n-1)\right]+\\ & +nf\left[\tilde{\gamma}(k-2n+1)\right].\end{aligned} \tag{9}$$

To short the notation, we define

$$\begin{aligned} f(\Lambda_0) &\equiv f\left[\tilde{\gamma}(k-2n)\right]\left[1+k(1-s)\right],\\ f(\Lambda_-) &\equiv f\left[\tilde{\gamma}(k-2n-1)\right],\\ f(\Lambda_+) &\equiv f\left[\tilde{\gamma}(k-2n+1)\right],\end{aligned}$$

where all Λ implicitly depend on γ. Thus, depending on the value of the synaptic strength, some the above terms can saturate, i.e. $f(\Lambda) = 1$. In fact, if we that $\gamma > \gamma_c$, then $f(\Lambda_+) = f(\Lambda_0) = 1$. Under this assumption, replacing the value at which the bifurcation happens in Eq.(11), we obtain

$$\gamma^{sat} = \frac{1-k(1-\alpha)}{(1-\alpha)-k(1-\alpha)(1-2\alpha)}, \tag{10}$$

which coincides very accurately with the point at which the activity becomes $s = 1$ in the sparse model, as observed in numerical simulations (see Fig. 2 in main text). Note that at the limit $k \to +\infty$ this coincides with the mean-field critical point, but for sparse networks, saturation occurs after γ_c. Note that the mean-field can be recovered again if we let all the three functions to be $f(\Lambda) = \Lambda$, i.e. none of them saturates.

3 Robustness of the results

Here we explore the robustness of the results and conclusions presented in the main text with respect to the modification of various modelling choices.

Changes in the transfer function

First we discuss modifications in the transfer function $f(\Lambda)$, that represents the probability of activation of a neuron with input Λ. The characteristics of the mean-field phase transition, depend on the particular election of this function. In mean-field approximation, $\langle f(\Lambda)\rangle \simeq f(\langle\Lambda\rangle) = f(\gamma(1-2\alpha)s)$ (see derivation in SI-1). Then, the equation for the activity is simply

$$\dot{s} = f(\gamma(1-2\alpha)s) - s, \tag{11}$$

which has fixed points satisfying $f(\gamma(1-2\alpha)s^*) = s^*$. While the position of the critical point γ_c is robust under changes of the transfer function, the stability of the fixed points may be altered. As a consequence, the shape of the mean-field phase diagram

strongly depends on the choice of the transfer function. Since, in mean-field approximation, the activity is bounded to lie in the interval $0 \leq \Lambda \leq 1$, it is possible to Taylor expand $f(\Lambda)$ around $\Lambda = 0$ (which is always a fixed point):

$$f(\Lambda) = a\Lambda + b\Lambda^2 + c\Lambda^3 + \dots \tag{12}$$

where a, b and c are un-specified parameters. This is often called a *Landau expansion* in the theory of critical phenomena[3]. Note that the linear term in Eq.(11) can be absorbed into the a term of Eq.(12), just shifting its value. The signs of the coefficients a, b, c... determine the behaviour of stable fixed points of the system. Fig.S1 shows the phase transition arising from different combinations of these coefficients. In particular, the rightmost figure shows the most realistic case in which there is a region of bistability with hysteresis.

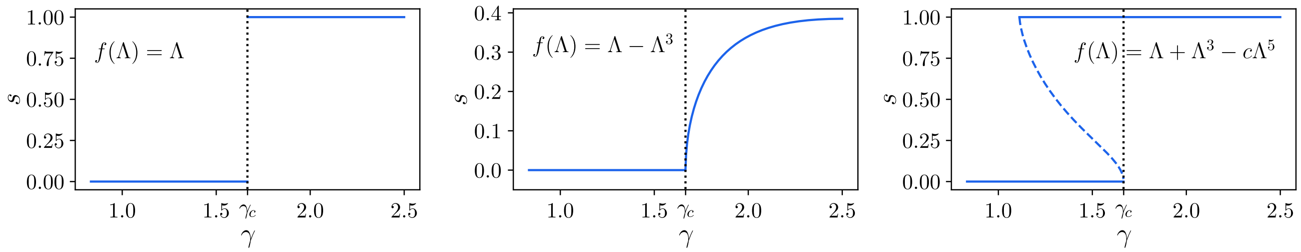

Figure S1. Mean-field phase diagram of Eq.(11) for different choices of the transfer function $f(\langle\Lambda\rangle) = f(\gamma(1-2\alpha)s)$. Depending on the signs of the coefficients chosen in the Landau expansion, we may observe discontinuous, continuous, or discontinuous with bistability types of bifurcations (the discontinuous blue line represents unstable fixed points). As illustrated in the main text in all cases an intermediate LAI phase emerges once sparse networks are considered..

Some of the key properties of these phase transitions persist even for sparse networks, where the LAI phase appears. We have computationally verified that the presence of the self-sustained LAI phase is not altered by the choice of $f(\Lambda)$; it exists both in the case in which the mean-field transition is continuous (see Fig.S2) and discontinuous with bistability and hysteresis (see Fig.S3). In particular, we wondered whether the asynchronous irregular spiking and other characteristic features of the LAI phase –as reported in the main text– are exclusive of the LAI phase or also appear in the active phase in the case in which this exhibits a low level of activity (e.g. in the case of a continuous phase transition, such as that of Fig.S1 central). Fig.S2) shows the coefficient of variation (CV) and time-lagged cross-correlation (CC), revealing that both of these quantities are approximately constant and large inside the LAI phase, but they decay quickly as soon as the coupling parameter γ enters the active phase. We conclude that the dynamical properties of the characteristic features of the LAI phase cannot be found in a regular active phase, even when this also displays relatively low activity.

In the case of the continuous mean-field transition, we can take advantage of the functional form of the hyperbolic tangent, in order to compute analytically an expression for the Jensen's force. If the response function $f(\Lambda)$ can be expanded in Taylor series (for $s > 0$), we can approximate the Jensen's stochastic force as

$$F(\Lambda) = \sum_{n=0}^{+\infty} \frac{f^{(n)}(0)}{n!} \left(\langle\Lambda^n\rangle - \langle\Lambda\rangle^n\right), \tag{13}$$

for positive inputs. The moments of the input distribution may be computed as shown in the previous Appendix, and thus it is possible to obtain an analytical approximation to the desired order n. If we use $f(\Lambda \geq 0) = \tanh\Lambda$, performing the expansion[1] we obtain that the first term that contributes to the Jensen's force is the one that corresponds to the third moment,

$$F(\Lambda) \equiv F(s) \simeq \frac{\gamma^3}{3}\alpha(1-\alpha)(1-2\alpha)ks(1-s)(1-2s). \tag{14}$$

Observe that in this case the Jensen's force vanishes at $s = 0, 1/2, 1$, in agreement with what we noticed numerically in the main text for the case of the linear piecewise function. In particular, let us also remark that –in the detailed-balanced case– where the mean-field term vanishes and the dynamics is given $\dot{s} = F(s)$, $F(s)$ is of the form [2] $F(s) \sim as - bs^2$, showing the continuous quiescent-active transition.

[1] Since we use $f(\Lambda < 0) = 0$, the response function is not analytic at the origin –hence, it cannot be Taylor expanded at 0. However, we assume that the expansion is formally valid for all $\Lambda > 0$. A similar calculation cannot be performed for the linear piecewise function, due to the saturation condition $f(\Lambda \geq 1) = 1$.

[2] Since $0 \leq s \leq 1$, the $+cs^3$ does not destabilize the system, and can be ignored.

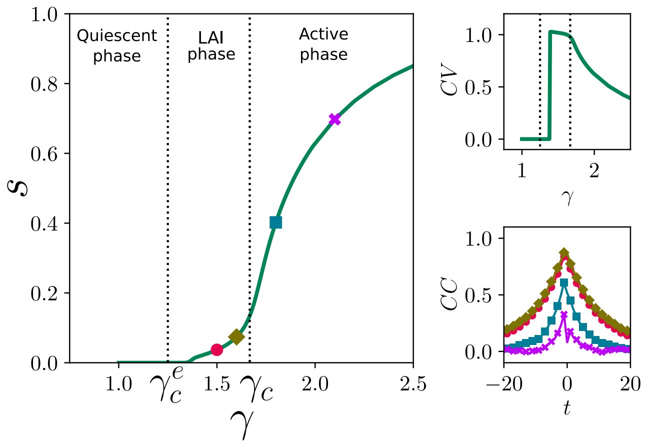

Figure S2. (a) Phase diagram for the model implementing a hyperbolic tangent (for $s > 0$ and 0 otherwise) as a transfer function in a hyper-regular network with $N = 16,000$ nodes and $k = 40$. The critical points, γ_c^e and γ_c, are marked with dotted lines. Observe the presence of an intermediate (LAI) phase, as well as an active phase that emerges after a continuous phase transition. (b) Coefficient of variation CV as a function of the coupling γ. Note that CV is approximately constant inside the LAI phase, where it takes a relatively large value, but its value decreases as soon as one enters either the quiescent or the active phase. (c) Time-lagged cross-correlations CC for the points marked with symbols in the panel (a). The maximum of the correlation starts decreasing as γ goes inside the active phase, meaning that excitation and inhibition become progressively decorrelated within the LAI phase.

In the case in which the mean-field phase transition is discontinuous with bistability, it is also pertinent to ask whether the emerging LAI phase is able to coexist with the active phase. In order to do that, we consider $f(\Lambda) = \Lambda + \Lambda^3 - c\Lambda^5$ and run simulations for different initial activities s_0 [3]. Results are presented in Fig.S3: there is coexistence between the LAI phase (with low but non-vanishing activity) and the active phase. Low initial activity values end up flowing to the LAI state, while higher values of s_0 drive the system to the active phase, with larger values of the activity, illustrating the bistability of the dynamics. When γ is increased, s slowly increases, until the activity value goes over the instability point (smaller than the mean-field value $\gamma_c = 1/(1-2\alpha) = 1.667$). Above this point, the LAI phase becomes unstable and the system jumps into the active phase. Thus the system exhibits bistability and hysteresis, between the LAI phase and the regular active one.

Changes in the network structure

Simulations for annealed and quenched hyper-regular networks show that both cases give the same results computationally, and that such results coincide with the analytic predictions. Thus, as analytical computations are exact only in the annealed case, the essence of the involved mechanism has nothing to do with the network specific structure and, thus, spectral analyses of the connectivity matrix[3,4] do not add much to the understanding of the noise-induced intermediate phase.

[3] This transfer function is similar to the truncated Taylor expansion of the hyperbolic tangent, changing signs of the non-linear term coefficients. The c term controls the width of the hysteresis cycle, and has been set to $c = 1/2$.

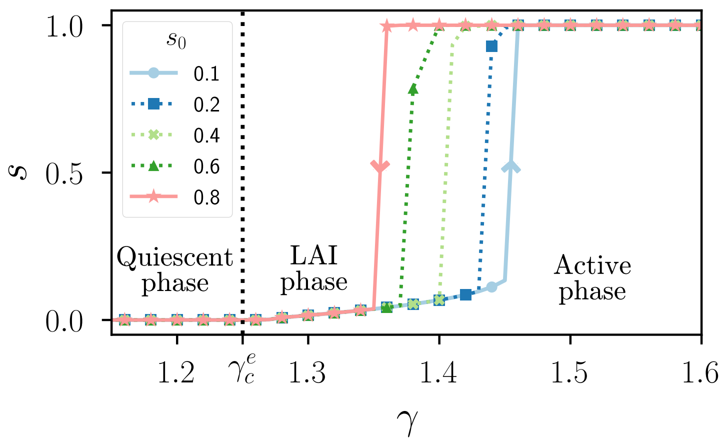

Figure S3. Phase diagram for a transfer function $f(\Lambda) = \Lambda + \Lambda^3 - \frac{1}{2}\Lambda^5$, for different initial states s_0 (see legend). The system displays bistability between the LAI phase and the active phase in a full interval of γ values. $N = 128000$ and $k = 15$.

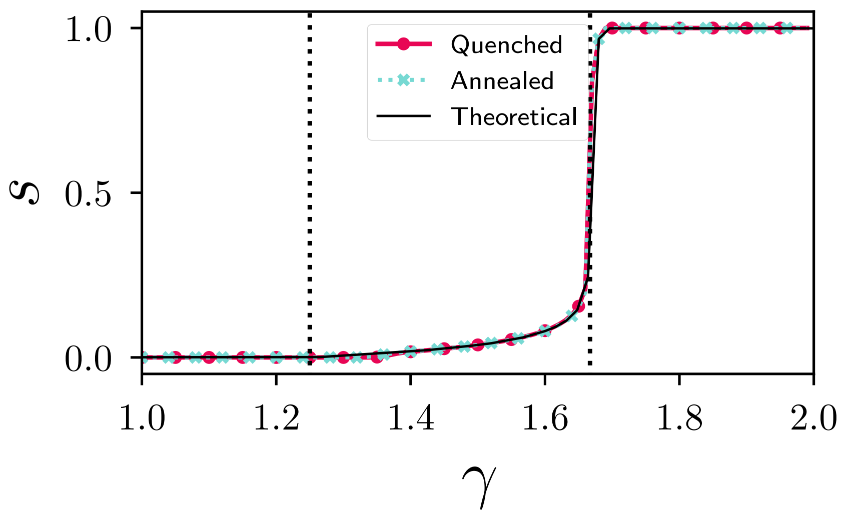

Figure S4. Comparison between annealed and quenched networks (simulation results marked with symbols) and the theoretical prediction for hyper-regular networks with connectivity $k = 40$ ad size $N = 16000$. The agreement between quenched and annealed hyper-regular networks is excellent (perfect within numerical accuracy), and the agreement between these two and analytical predictions is also excellent for sufficiently large network sizes.

We verified computationally that the main results also emerge in more irregular networks. In particular, simulations on Erdős-Rényi networks also reveal the emergence of a LAI phase, as shown in Fig.S5. Similarly, considering a Gaussian distribution of weights with variance $\sigma^2 = 1$ (rather than ± 1), does not affect either the existence of a LAI phase (see Fig.S5).

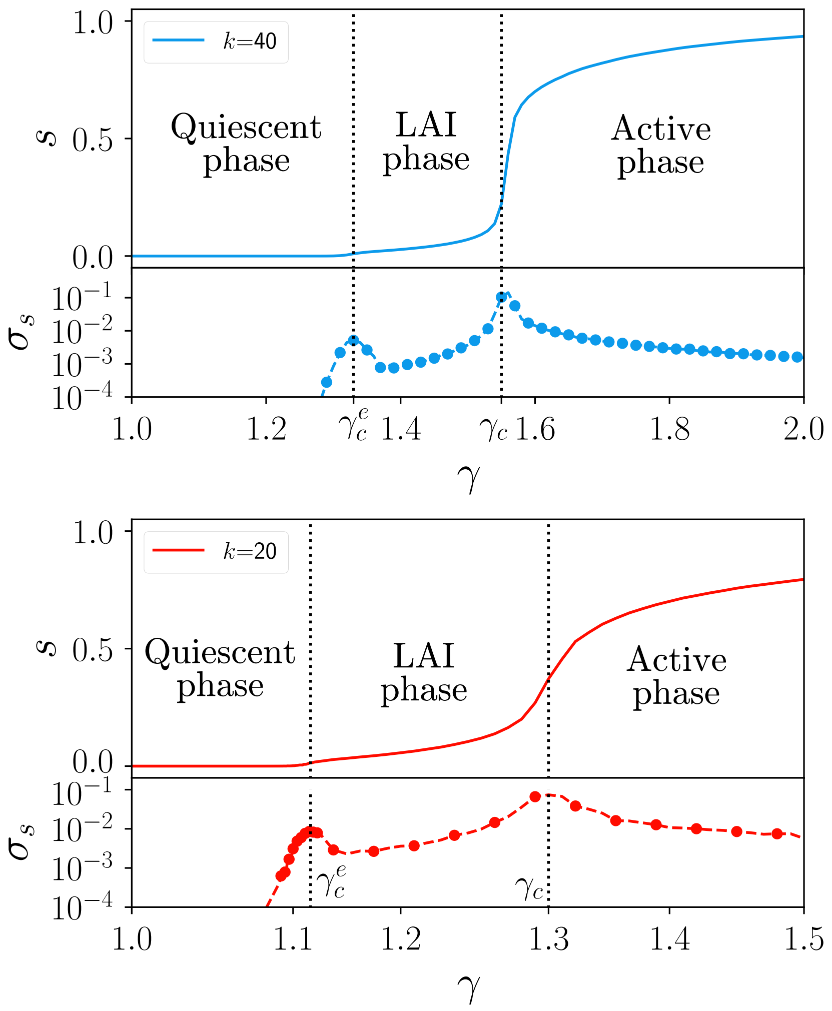

Figure S5. (Up) The LAI phase emerges also in (non-regular) unweighted Erdős-Rényi networks with mean connectivity $k = 40$ and $N = 16000$ as well as in (Down) Erdős-Rényi networks with a Gaussian weight distribution, $k = 20$ and $N = 16000$.

We have checked the change of the coefficient of variation when the model runs on an Erdos-Renyi network instead of the hyperregular one. As shown in Fig. S6, the CV of the Erdos Renyi network is slightly larger, meaning that increasing the heterogeneity can increase the irregularity of the spiking neurons. The results, however, are still far away from the values observed in experiments as discussed in the main text.

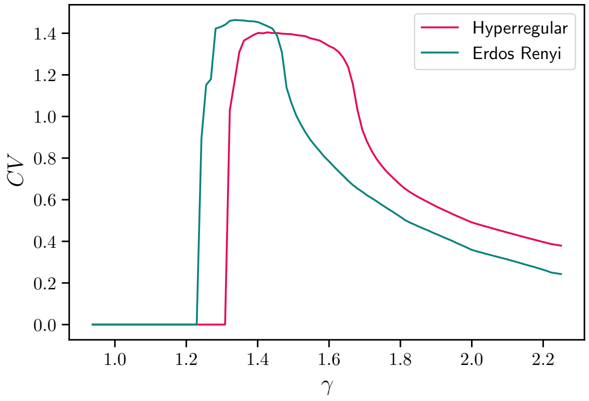

Figure S6. Comparison between the CV of hyperregular and Erdos-Renyi networks. Both have a CV higher than 1 in the LAI phase; the coefficient of the Erdos Renyi network is slighly larger. Simulations were run in networks of size $N = 16000$, with $k = 20$. After relaxation time, the system was simulated for $t = 10^3$ time units.

Correlation among subpopulations

In the main text, we presented the excitation-inhibition lag as one of the indicators of the asynchronous irregular phase. Here we briefly study the correlations among all the subpopulations. In order to do that, we simulate our model for $t = 10^3$ in a hyperregular network with $N = 16000$, $k = 20$, and store the data of each individual neuron spike. Then, we compute the total activity of a subpopulation, and the cross-correlation with the activity of another subpopulation. To compute the E-E correlation, we divide the excitatory population in two halves. The same is done for the inhibitory population. Results are summarized in Figure S7.

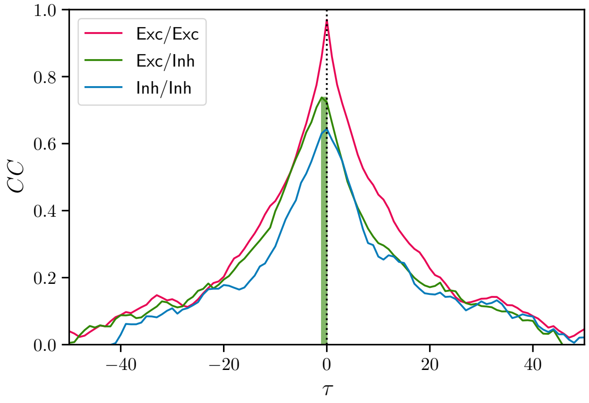

Figure S7. Cross-correlation among the different subpopulations in the system. Both the E-E and I-I correlations peak at $\tau = 0$, meaning that the series are very correlated when there is no lag between them. However, results for E-I correlation show a slight lag between the series, as in the simulations presented in main text. The E-I lag has been remarked with a light color fill.

Cross-correlations peaks at the origin for both the E-E and I-I populations. This means that the excitatory population behave collectively as a whole; the same applies to the inhibitory population. However, there is a slight E-I lag present between the two populations, meaning that the inhibitory population is behaving collectively, but spiking right after the excitatory does. As discussed in the main text, this is a typical feature in asynchronous irregular states, and has been found both in models and experiments.

4 Experimental measurement of Jensen's force

In order to explicitly observe and measure Jensen's forces in the laboratory, we propose the following (preliminary) experimental protocol:

(i) Consider observations of neuronal activity in the asynchronous state such as those already in the existing literature. Measure the average network activity $s(t)$ as a function of time (in discrete time bins) and determine the value $s(t+1)$ as a function of the activity at the preceding timestep $s = s(t)$ for all possible observed values of s and average across the steady-state time series to obtain good statistics. This procedure provides us with an empirical estimation of the averaged response transfer function as $s(t+1) = \langle f(\Lambda) \rangle_{exp}$ (as easily derived from Eq.(3) in the main text). Let us remark that there exist publicly available empirical datasets (e.g. for the rat and cat cortex; see[5] and refs. therein) that can be used for this purpose, though better statistics including many more neurons and longer observations times would be highly desirable.

(ii) Extract individual neurons from the same tissue under study and determine empirically *in vitro* their associated transfer function $f_{exp}(\Lambda)$ (where Λ is the input). We believe that this is experimentally feasible as similar measurements have been already successfully performed[6,7]. If, in the experiments, the responses of diverse neurons are different, a sort of averaged neuron response should be constructed as a proxy for $f_{exp}(\Lambda)$[6].

(iii) Measuring the membrane potential, it should be possible to estimate the averaged input received by a single neuron in the network, $\langle \Lambda \rangle$ and using the result of (ii) it should be possible to compute $f_{exp}(\langle \Lambda \rangle)$.

(iv) The state-dependent Jensen's force is then determined using Eq.(8) in the main text, i.e.

$$F(\Lambda) = \langle f(\Lambda) \rangle_{exp} - f_{exp}\left(\langle \Lambda \rangle\right). \tag{15}$$

The theory presented here predicts a non-linear and non-monotonic behavior for $F(\Lambda)$, similar to that of the inset of Fig.4. Note also that the theoretical prediction could be refined and made more specific by implementing in the theoretical model the empirically determined transfer function, $f_{exp}(\Lambda)$. This change might hinder analytical calculations, but would be straightforward to implement in computational analyses of our simple model, that would lead to a specific prediction for the Jensen's force in the experimental setup. In any case, for states of low activity the difference between the averaged response within the network $\langle f_{exp}(\Lambda) \rangle$ is expected –according to the theory developed here– to be larger than the response of individual neurons to the average activity $f_{exp}\left(\langle \Lambda \rangle\right)$, i.e. there should be a repulsive stochastic force, inducing fluctuating states of low-activity, and this should be observed in the experiments.

We leave this programme –which is very likely to need refinements to account for a number of potential experimental pitfalls, such as statistical error from subsampling, time-binning ambiguities, heterogeneous response of individual neurons, etc.– for future research and as an open challenge for experimentalists.

References

1. Van Vreeswijk, C. & Sompolinsky, H. Chaos in neuronal networks with balanced excitatory and inhibitory activity. *Science* **274**, 1724–1726 (1996).
2. Barral, J. & Reyes, A. D. Synaptic scaling rule preserves excitatory–inhibitory balance and salient neuronal network dynamics. *Nat. Neurosci.* **19**, 1690 (2016).
3. Binney, J. J., Dowrick, N. J., Fisher, A. & Newman, M. E. *The Theory of Critical Phenomena* (Oxford University Press, Oxford, 1993).
4. Chen, X. & Dzakpasu, R. Observed network dynamics from altering the balance between excitatory and inhibitory neurons in cultured networks. *Phys. Rev. E* **82**, 031907 (2010).
5. Zierenberg, J., Wilting, J. & Priesemann, V. Homeostatic plasticity and external input shape neural network dynamics. *Phys. Rev. X* **8**, 031018 (2018).
6. Wolfart, J., Debay, D., Le Masson, G., Destexhe, A. & Bal, T. Synaptic background activity controls spike transfer from thalamus to cortex. *Nat. Neurosci.* **8**, 1760 (2005).
7. La Camera, G. *et al.* Multiple time scales of temporal response in pyramidal and fast spiking cortical neurons. *J. Neurophysiol.* **96**, 3448–3464 (2006).
